# A Novel *CCM2* Missense Variant Caused Cerebral Cavernous Malformations in a Chinese Family

**DOI:** 10.3389/fnins.2020.604350

**Published:** 2021-01-05

**Authors:** Guoqing Han, Li Ma, Huanhuan Qiao, Lin Han, Qiaoli Wu, Qingguo Li

**Affiliations:** ^1^Department of Neurosurgery, Tianjin Huanhu Hospital, Tianjin, China; ^2^Department of Preventive Dentistry, School of Stomatology, Tianjin Medical University, Tianjin, China; ^3^Tianjin Key Laboratory of Brain Science and Neural Engineering, Academy of Medical Engineering and Translational Medicine, Tianjin University, Tianjin, China; ^4^Running Gene Inc., Beijing, China; ^5^Tianjin Neurosurgical Institute, Tianjin Huanhu Hospital, Tianjin, China

**Keywords:** familial cerebral cavernous malformation, *CCM2*, missense variant, chinese family, susceptibility-weighted imaging

## Abstract

Cerebral cavernous malformations (CCMs) are common vascular malformations in the central nervous system. Familial CCMs (FCCMs) are autosomal dominant inherited disease with incomplete penetrance and variable symptoms. Mutations in the *KRIT1*, *CCM2*, and *PDCD10* genes cause the development of FCCM. Approximately 476 mutations of three CCM-related genes have been reported, most of which were case reports, and lack of data in stable inheritance. In addition, only a small number of causative missense mutations had been identified in patients. Here, we reported that 8/20 members of a Chinese family were diagnosed with CCMs. By direct DNA sequencing, we found a novel variant c.331G > C (p.A111P) in exon 4 of the *CCM2* gene, which was a heterozygous exonic variant, in 7/20 family members. We consider this variant to be causative of disease due to a weaken the protein–protein interaction between *KRIT1* and *CCM2*. In addition, we also found the exon 13 deletion in *KRIT1* coexisting with the *CCM2* mutation in patient IV-2, and this was inherited from her father (patient III-1H). This study of a Chinese family with a large number of patients with CCMs and stable inheritance of a *CCM2* mutation contributes to better understanding the spectrum of gene mutations in CCMs.

## Introduction

Cerebral cavernous malformations (CCMs) are the second most prevalent type of vascular malformation characterized by clustered enlarged capillary-like vessels and the absence of intervening neural tissue in the central nervous system (Rigamonti et al., 1988). The diameters of developmental venous anomalies range from a few millimeters to several centimeters (Rigamonti et al., 1988; Gil-Nagel et al., 1995). With an incidence of 0.1–0.5%, CCMs represent 10 to 20% of all cerebrovascular malformations (Flemming et al., 2017; Scimone et al., 2017). Clinical manifestations most often occur between 20 and 30 years old. These patients most commonly experience seizures (40–70%), focal neurologic deficits without intracranial bleeding (25–50%), non-specific headaches (10–30%), and intracranial hemorrhage (25–32%) (Akers et al., 2017). However, approximately 40% of all CCM patients are asymptomatic (Labauge et al., 2007). The majority of CCMs are sporadic and comprise a single lesion, while the rest (approximately 20%) are familial CCMs (FCCMs), which are caused by autosomal dominant inherited mutations and are usually associated with multiple lesions (Zafar et al., 2019).

In FCCMs, three related genes, namely *CCM1* (Krev interaction trapped-1, *KRIT1*), *CCM2* (*MGC4607*), and *CCM3* (programmed cell death protein 10, *PDCD10*), have been reported (Dupre et al., 2003; Bergametti et al., 2005). According to the Human Gene Mutation Database (HGMD) (updated through December 2019), there are 476 germline mutations of the three genes that have been described as causative mutations of CCMs (Scimone et al., 2018). A large majority of causative mutations include non-sense, frameshift, and splicing mutations, commonly resulting in a premature stop codon and dysfunctional mRNA (Zafar et al., 2019). However, only a small number of causative missense mutations have been identified in patients, and most of them lead to abnormalities in splicing (Riant et al., 2013; Bergametti et al., 2020).

Herein, we reported a large Chinese family with multiple members having CCMs; two patients were seen in our institute, and we summarized the clinical and radiological features of 20 members from this family. We screened CCM-related genes through whole-exome sequencing (WES) and identified a novel missense variant (c.331G > C) in *CCM2* that caused at least seven cases of CCMs; we also identified the exon 13 deletion in *KRIT1* coexisting with the *CCM2* mutation in patient IV-2 and her father (patient III-1H). This study contributes to better understanding the spectrum of mutations of FCCM.

## Materials and Methods

### Whole-Exome Sequencing

Peripheral blood samples were collected from members of the family and then sent to Running Gene Inc (Beijing, China) for sequencing. WES was performed and included the targeted *KRIT1*, *CCM2*, and *PDCD10* genes. DNA samples were extracted by a DNA Isolation Kit (AU1802, BioTeke, China), concentrated using a Qubit dsDNA HS Assay Kit (Q32851, Invitrogen, United States), purified, and PCR amplified with Agencourt AMPure XP beads. Targeted DNA samples were sequenced on HiSeq X10 (Illumina, San Diego, United States). All variants were filtered first against the 1000 Genomes Project database for a minor allele frequency (MAF) ≥ 1% and ExAC hom AC ≥ 3. The obtained variants were further selected according to co-segregation, genetic model, and MAF < 1% in three databases (1000 Genomes Project_EAS, ExAC, gnomAD_EAS).

According to the American College Medical Genetics and Genomics (ACMG) guidelines, mutations was analyzed to assess the risk of pathogenicity (Richards et al., 2015). Sanger sequencing was used to validate the family segregation of mutations. The variants were identified as novel when they were not reported in previous literatures or were not found in the following public databases: (1) CCM mutation database^1^ (2) dbSNP at the National Center for Biotechnology Information (NCBI)^2^; (3) ClinVar at NCBI^3^, and (4) HGMD^4^ (Huang et al., 2016).

## Results

### Case Presentation

**FIGURE 1:**
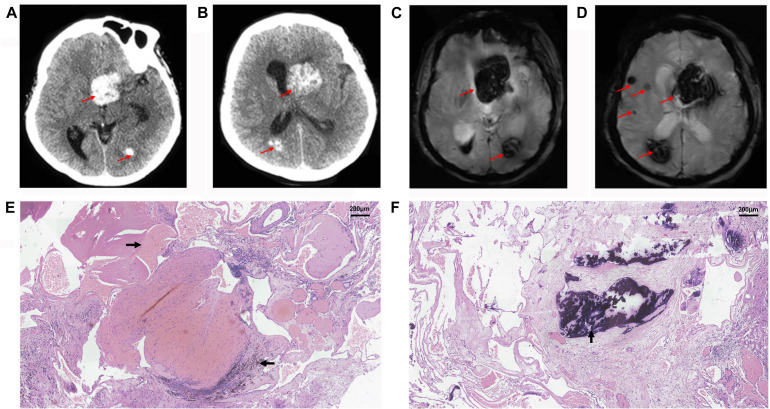
Radiological and histopathological information of patient II-1. CT **(A,B)** and GRE T2-weighted MRI **(C,D)** showed multiple cavernous malformation lesions across the left lateral ventricle and bilateral cerebral hemisphere in the proband, patient II-1. The red arrows show the location of lesions. Histopathological examination revealed vascular malformation associated with bleeding (**→**), calcification (**↑**), and iron deposits (**←**) in the proband, patient II-1, at **(E)** ×100, and **(F)** ×100.

The proband (II-1) was a 60-year-old woman who had complained of an inability to walk for 10 years, and was admitted to our hospital because of hypophrasia, and loss of consciousness for 2 days. The left lateral ventricle and subcortical regions of the cerebral hemispheres were showed multiple high-intensity patchy calcifications or bleeding from computed tomography (CT) findings (Figures 1A,B). Gradient-recalled echo (GRE) T2-weighted magnetic resonance imaging (MRI) showed a large number of dark spots, and mass of different sizes distributed in the left lateral ventricle, bilateral cerebral hemisphere, and cerebellum (Figures 1C,D), which indicated multiple cavernous malformations. The patient received an operation on the left lateral ventricle lesion. However, her symptoms were not improved shortly. Conversely, she suffered the complications of hypothalamic diabetes insipidus, pulmonary infection, and loss of consciousness for a long time. Pathological observations showed vascular malformations (mixed type) associated with bleeding, calcification, and iron deposits (Figures 1E,F). The Ki-67 proliferation rate was 2.6%. After nearly a month of treatment, she gradually regained consciousness.

**FIGURE 2:**
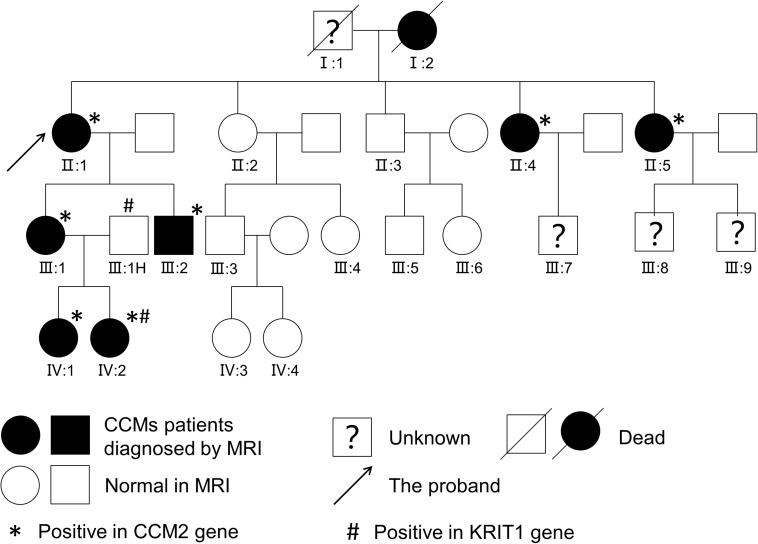
Pedigree of a Chinese family. Affected patients were diagnosed with CCMs upon T2-weighted MRI or SWI of the brain.

Considering that multiple CCMs usually occur in FCCMs, we present the family history and brain MRI examination results. Surprisingly, we found high morbidity (40%, 8/20) in four generations. Here, we display the pedigree of the Chinese family in Figure 2, and all of the detailed clinical information is listed in Table 1.

**TABLE 1:** Clinical data and genotypes of the members of a Chinese family with CCMs.

Patient	Gender	Age at diagnosis	Symptoms	Localization	Treatment	Lesions single or multiple	Other combined lesion	Variant nucleotide	Variant gene	Coding amino acid change
I-2	F	78	No data available	Bilateral temporal, parietal, basal ganglia, right cerebellum, bilateral paraventricular	No	Multiple	Meningioma at right parietal	Not available	Not available	Not available
II-1	F	60	Inability to walk, hypophrasia, loss of consciousness	Bilateral frontal, parietal, occipital, paraventricular, right cerebellum	Surgery	Multiple	Meningioma at right frontal			
II-4	F	39	Asymptomatic	Bilateral frontal, temporal, parietal, right, basal ganglia	No	Multiple	Lesions in T1 Spinal cord			
II-5	F	40	Dizziness, seizures	Bilateral frontal, right parietal, right paraventricular	Surgery	Multiple		c.331G (>C	CCM2	p.Ala111Pro
III-1	F	18	Asymptomatic	Right frontal, right parietal, brainstem	No	Multiple				
III-2	M	25	Asymptomatic	Left temporal	No	Single				
IV-1	F	6	Headache	Left temporal, left triangle area of lateral ventricle	No	Multiple	None			
IV-2	F	9	Asymptomatic	Left triangle area of lateral ventricle	No	Single		c.331G (>C &and Exon 13del	*CCM2* and KRIT1	p.Ala111Pro Not available
III-1H	M	35	Asymptomatic	None	No	None		Exon 13del	KRIT1	Not available

**FIGURE 3:**
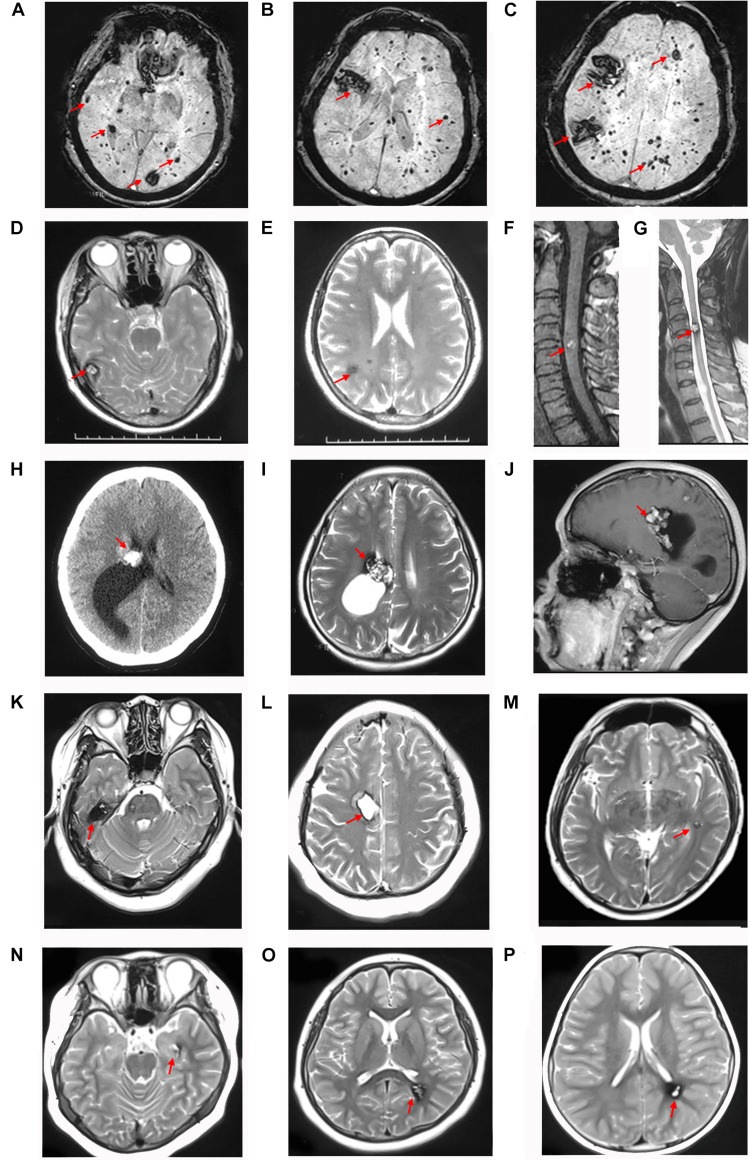
CCM lesions diagnosed through CT, T2-weighted MRI and SWI. **(A–C)** SWI of patient I-2 showed hundreds of lesions distributed across the cerebral hemisphere and cerebellum. T2-weighted MRI of patient II-4 showed multiple CCMs in the right temporal lobe **(D)**, frontal and parietal lobes **(E)**, and spinal cord **(F,G)**. CT **(H)**, T2-weighted **(I)**, and contrast MRI **(J)** of patient II-5 showed lesions with a “popcorn” appearance located in the right lateral ventricle and frontal-parietal lobe with asymmetrical ventricles. **(K,L)** T2-weighted MRI of patient III-1 showed lesions surrounded by a dark rim of hemosiderin on the right frontal and temporal lobes. **(M)** T2-weighted MRI of patient III-2 showed a lesion located on the left temporal lobe. **(N,O)** T2-weighted MRI of patient IV-1 showed lesions on the left temporal lobe and triangle area of the lateral ventricle. **(P)** T2-weighted MRI of patient IV-2 showed a lesion located in the left triangle area of the lateral ventricle. The red arrows show the CCM lesions.

Patient I-2, aged 83 years, was diagnosed with CCM approximately 6 years ago. Susceptibility-weighted imaging (SWI) of the brain showed hundreds of dark spots and mass of different sizes distributed in the cerebral hemisphere and cerebellum (Figures 3A–C) combined with meningioma at the right parietal lobe. The diameters of the CCM lesions ranged from 1 mm to 3 cm. She didn’t receive any treatment and unfortunately died 1 month before we conducted this study. Patient II-5 was a 41-year-old woman, showing dizziness and epileptic seizures for 3 days. Non-contrast CT revealed a high-intensity calcification or bleeding located in the right lateral ventricle with asymmetrical ventricles (Figure 3H). T2-weighted gradient echo (GE) sequences showed that multiple CCM lesions were distributed in the right lateral ventricle and across the hemisphere without contrast (Figures 3I,J). Patient II-5 underwent resection of the right lateral ventricle lesion. The seizures in this patient subsided, but this treatment caused hemiplegia of the left limb. Patient IV-1, aged 13 years, complained of intermittent headaches and was diagnosed with CCM since she was 6 years old. T2-weighted MRI showed lesions on the left temporal and triangular areas of the lateral ventricle (Figures 3N,O). Patient I-1 died several years ago, and the family had lost contact with patients III-7, III-8, and III-9. Therefore, their MRI data were not available. Patients II-4 (Figures 3D–G), III-1 (Figures 3K,L), III-2 (Figure 3M), and IV-2 (Figure 3P) were asymptomatic.

### Mutational Analysis

**FIGURE 4:**
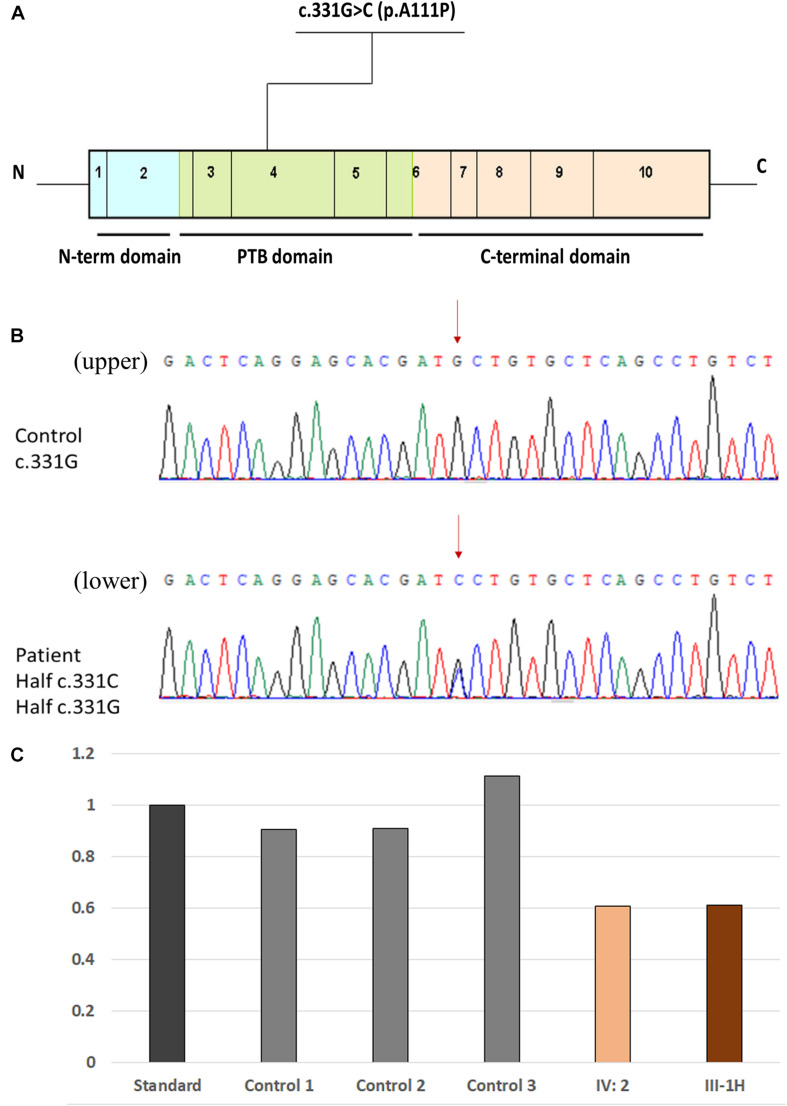
Location of the novel missense mutation. **(A)** Schematic representation of the genetic structure and protein domains of *CCM2*. The novel missense mutation c.331G > C (p.A111P) is located in the PTB domain of exon 4 in the *CCM2* gene. **(B)** A novel heterozygous missense mutation was detected by Sanger sequencing. (Upper panel) The normal sequences of the *CCM2* gene. (Lower panel) The genetic variant of c.331G > C chr7-45104104 (p.A111P). The affected nucleotide is indicated with red arrows. **(C)** Comparison of the DNA-related content in exon 13 in *KRIT1.* Data among the standard, patient IV-2, and patient III-1H (father of patient IV-2) were shown.

WES was performed on 15 members of a Chinese family, except for patients I-1 and I-2 as both were dead and patients III-7, III-8, and III-9 (lost contact with the family). Detailed results are also listed in Table 1. A heterozygous variant c.331G > C (Chr7:45104104) in exon 4 of the *CCM2* gene (NM_031443, NP_113631) was detected in 7/20 family members (Figures 4A,B). This variant caused the 111st amino acid alanine to be replaced with a proline (p.A111P), and native Ala111 residue is located in the phosphotyrosine binding (PTB) domain as a putative phosphoinositide binding site (PM1). Variant c.331G > C is absent from public databases such as gnomAD, 1000 Genomes, ExAC (PM2), and it is also a novel mutation that has not been reported in the dbSNP, HGMD, ClinVar, and CCM mutation databases or in the previous literature. The variant co-segregated with CCMs in seven affected family members (PP1), and it is predicted as deleterious in multiple computational software (PP3), including MutationTaster2 (disease causing, probability: 1.000), SIFT (damaging, 0.008 < 0.05), PROVEAN (deleterious, −2.65 < −2.5), and PolyPhen-2 (probably damaging, probability: 0.999). Thus, the variant c.331G > C (p.A111P) was considered to be likely pathogenic, according to ACMG standards and guidelines (Richards et al., 2015).

The other variant, a deletion in exon 13 of *KRIT1* (NM_194456), was detected in the proband’s second granddaughter (IV-2), who was asymptomatic (Figure 4C). To ensure the source of this *KRIT1* gene mutation, we used WES to examine the DNA from patient IV-2’s father (patient III-1H) and found the identical deletion; however, patient III-1H had a normal MRI scan. The variant was a heterozygous genomic deletion mutation and has been reported in the HGMD as associated with CCM lesion development through a splicing impairment (Mondejar et al., 2014).

**FIGURE 5:**
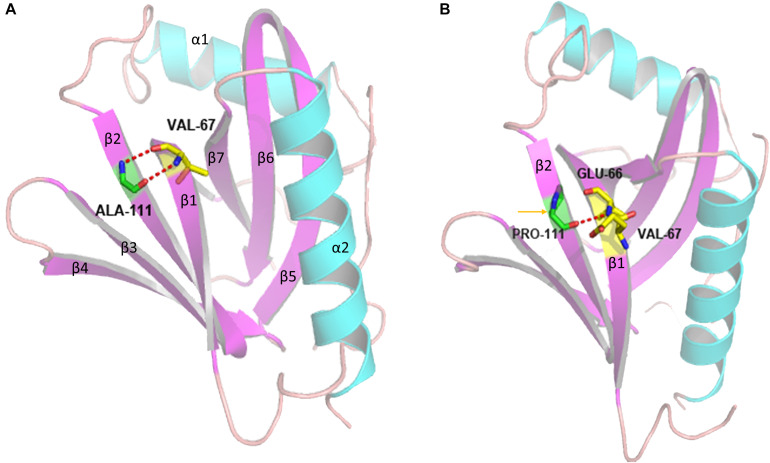
Three-dimensional structure of the PTB domain generated by PyMOL software. The panel shows the overlap between the wild-type **(A)** and mutated **(B)** tertiary protein structures. p.A111P: superimposed structures of the malcavernin protein with the native Ala111 residue and Pro111 residue substitution. The yellow arrow indicates the region where the A111P substitution resides, and the dotted red lines indicate the alteration in the intramolecular H bonds in the area of the substitution.

We used an *in silico* approach to test the effect of the novel c.331G > C variant of the *CCM2* gene. A structural comparison of the wild-type and mutated proteins was performed by PyMOL software^5^ (Figure 5). To predict the secondary structure of the polypeptide, we used an extensible molecular modeling system, the UCSF Chimera^6^ tool. From the *in silico* analysis, we found that the native Ala111 residue in the PTB domain (located in the beta 2 strand) of the malcavernin protein interacted with Val67 (located in the beta 1 strand) through two intramolecular hydrogen (H) bonds. Once the Pro111 mutant was superimposed, a change in the number, and position of the intramolecular H bond of the PTB domain could cause an alteration in the folding at position 111.

## Discussion

In the 1980s, (Hayman et al., 1982) and (Rigamonti et al., 1987) suggested that FCCMs may be an autosomal dominant inherited disease with incomplete penetrance through analyzing the pedigree of an affected family. Dubovsky et al. (1995) mapped the *CCM1*/*KRIT1* gene in 1995 as it had a high prevalence rate in Hispanic families from southwestern United States. Craig et al. (1998) analyzed 20 non-Hispanic CCM families and identified and named two new CCM-related genes, *CCM2*, and *CCM3*/*PDCD10*. Previous studies have shown that the prevalence of mutations ranges from 70∼90% to 16∼60% in familial and sporadic CCMs, respectively (Spiegler et al., 2014; Merello et al., 2016), in which the *KRIT1*, *CCM2*, and *PDCD10* genes account for 60∼65%, 18∼19%, and 16∼22%, respectively (Riant et al., 2013; Mondejar et al., 2014; Spiegler et al., 2014). *KRIT1* mutations usually have a favorable clinical prognosis, while *PDCD10* mutations seem to show a more severe phenotype characterized by recurrent intracranial hemorrhage at a younger age (Shenkar et al., 2015).

In our study, seven members of the family carried the missense mutation c.331G > C, and all of them showed lesions on an MRI scan. Members without this mutation did not have lesions on an MRI scan. Clinical penetrance was estimated to be nearly 88% for *KRIT1*, 100% for *CCM2*, and 63% for *PDCD10* (de Vos et al., 2017). And *CCM2* is the only gene which was reported to cause 100% radiological changes, which means that the offspring have a 50% risk of developing the CCM disease if one of the parents carries the c.331G > C mutation. The majority of patients (7/8) in this study were female, suggesting that *CCM2* had a higher incidence in women. The ages at diagnosis ranged from 6 to 78 years old, and the median age of onset was 32 years old. Among the affected members (8/20), 3 patients complained of clinical symptoms. Patient IV-1 had a headache, while others were admitted due to focal nerve dysfunctions. Most (5/8) were asymptomatic and diagnosed only by an MRI examination. Patients with CCMs have heterogeneous phenotypes. Studies focused on genotype–phenotype correlations found a high variability of symptoms, even in patients belonging to the same family, and harboring the same mutation (Scimone et al., 2018). Heterogeneity of clinical manifestations and onset age were considered to be associated with the incomplete penetrance of the disease (Battistini et al., 2007).

Multiple lesions frequently occur in FCCMs and can be distributed in both the supratentorial and infratentorial regions. Considering a two-hit hypothesis for CCM pathogenesis, the different locations of lesions may be explained by the stochastic nature of somatic mutations (Akers et al., 2009). Among the patients with MRI data, two had a single brain lesion (III-2 and IV-4), and one had a lesion in the spinal cord (II-4), implying that even a single lesion, or a spinal lesion can be associated to the FCCMs, and the patients should be advised to a genetic counseling service (Cigoli et al., 2015).

Three patients (I-2, II-1, and II-4) also had other diseases, such as meningioma, suggesting that there may be some similarities in the development of the disease. In addition, we found a declining trend in the number of CCM lesions and complicated diseases in the four generations. The reason is still unclear, and it may be associated with the patients’ self-repair functions, which need to be further explored.

The proteins KRIT1, malcavernin, and PDCD10 compose a ternary complex that is involved in maintaining cell-cell junctions and cell-extracellular matrix adhesion, regulating the proliferation, and apoptosis of endothelial cells (Zafar et al., 2019). In the structure of the complex, malcavernin contains a PTB domain that mediates the interaction with KRIT1 and PDCD10 (Zawistowski et al., 2005). CCM2 acts as a bridge between KRIT1 and PDCD10 and interacts with the juxtamembrane region of the TrkA receptor tyrosine kinase, mediating TrkA-induced death in diverse cell types (Harel et al., 2009).

The *CCM2* gene encodes malcavernin, a 444 amino acid protein that contains an N-terminal flexible loop of 60 residues, a PTB domain (encoded by residues 60 to 220), and a harmonin homology domain (HHD) at its C-terminus (residues ∼220 to 444) (Fisher et al., 2013, 2015). The novel missense variant c.331G > C (p.A111P) that we identified is located in the PTB domain. Thus, we considered that the c.331G > C variant might cause the pathogenesis of FCCM through altering the structure and even destroying the function of the PTB domain.

From the *in silico* analysis, we found that c.331G > C (p.A111P) altered the number and position of intramolecular H–H bonds in the PTB domain. According to the pathogenic missense variants reported in the literature (Fisher et al., 2015; Bergametti et al., 2020), all missense variants changed the original amino acid to either a proline (9/11) or an arginine (2/11). Proline was usually found in some tight turns in protein structures where the polypeptide chain must change direction but was rarely involved in active or binding sites of the protein. Proline is also rarely found in α-helices and β-sheets, as it would reduce the stability of such structures, while arginine is a basic, polar amino acid that is positively charged and very hydrophilic (Bergametti et al., 2020). Furthermore, co-immunoprecipitation experiments found an absence of some amino acids located in the PTB domain KRIT1 could cause the loss of the interaction between KRIT1 and CCM2 (Fisher et al., 2015; Bergametti et al., 2020).

In addition, a deletion of exon 13 in *KRIT1* was detected in patient IV-2. This variant was a heterozygous genomic deletion and had been reported in a previous study to be associated with CCM lesion development through splicing impairment (Mondejar et al., 2014). Patient IV-2 harbored two mutations in the CCM gene, both of which could cause CCMs. However, the patient has not shown any manifestations to date and presented with only a single lesion. To estimate the origins of the two mutation sites, we further sequenced the DNA from her father (patient III-1H), who had a normal MRI result. Coincidentally, we found that her father (patient III-1H) also had the deletion of exon 13 in *KRIT1*, indicating that patient IV-2 inherited a causative gene from each parent. Both mutations were located on chromosome 7 (*KRIT1* was in the long arm, while *CCM2* was in the short arm). According to the penetrance of the *KRIT1* and *CCM2* genes, the predicted incidence rate of the offspring of patient IV-2 is 94%, but potential offspring have a 100% chance of carrying a causative gene mutation. Although no obvious symptoms were observed in this young patient, long-term follow-up is required to understand the ultimate pathogenicity of the relevant genes.

## Conclusion

We reported a Chinese family with four generations carrying the c.331G > C variant in exon 4 of *CCM2*, and this is a novel missense variant that causes CCMs. The novel variant in the *CCM2* gene showed autosomal dominant heredity and had 100% neuroradiological penetrance. This study of a family with a large number of patients with CCMs and stable inheritance of a *CCM2* mutation contributes to better understanding the spectrum of gene mutations in CCMs. We consider this variant to be causative of disease, but additional immunoprecipitation experiments will be needed to evaluate its pathogenesis. Specifically, patient IV-2 carries two causative mutations, including exon 13 deletion in *KRIT1* and c.331G > C in *CCM2*, with approximately a 100% predicted incidence rate in offspring. More advice on prenatal guidance and long-term follow-up are needed.

## Data Availability Statement

The datasets presented in this study can be found in online repositories. All the whole-exome sequencing cDNA (Illumina) data from this study have been submitted to the GenBank of NIH genetic sequence database (https://www.ncbi.nlm.nih.gov/WebSub/?form=history&tool=genbank) under accession number PRJNA639922.

## Ethics Statement

The studies involving human participants were reviewed and approved by the Research Ethics Committee of Tianjin Huanhu Hospital. Written informed consent to participate in this study was provided by the participants’ legal guardian/next of kin. Written informed consent was obtained from the individual(s), and minor(s)’ legal guardian/next of kin, for the publication of any potentially identifiable images or data included in this article.

## Author Contributions

GH and QL conceived the study. GH, LM, and HQ performed the clinical evaluation. QW performed the imageological assessment and MRI examination of patients. LH collected clinical information and also contributed to the sequencing, validation and analysis of novel mutations. GH and LH polished the figures and tables. GH drafted the manuscript. LH, HQ, and QL edited the manuscript. All authors read and approved the final manuscript.

## Conflict of Interest

LH is employed by Running Gene Inc., Beijing, China. The remaining authors declare that the research was conducted in the absence of any commercial or financial relationships that could be construed as a potential conflict of interest.

## Footnotes

1 https://www.angioma.org/

2 http://www.ncbi.nlm.nih.gov/snp/

3 https://www.ncbi.nlm.nih.gov/clinvar/

4 http://www.hgmd.org/

5 https://pymol.org/2/

6 http://www.cgl.ucsf.edu/chimera/download.html
